# Mention of ethical review and informed consent in the reports of research undertaken during the armed conflict in Darfur (2004–2012): a systematic review

**DOI:** 10.1186/s12910-019-0377-7

**Published:** 2019-06-13

**Authors:** Ghaiath Hussein, Khalifa Elmusharaf

**Affiliations:** 1 Doctoral Researcher, Medicine, Ethics, Science and Humanities (MESH), University of Birmingham, School of Public health, Birmingham, B15 2TT UK; 2 Senior Lecturer in Public Health, Graduate Entry Medical School, University of Limerick, Limerick, Ireland

**Keywords:** Research ethics, Humanitarian ethics, Non-governmental organizations, Public health ethics, Developing countries

## Abstract

**Background:**

Armed conflict in Darfur, west Sudan since 2003 has led to the influx of about 100 international humanitarian UN and non-governmental organizations to help the affected population. Many of their humanitarian interventions included the collection of human personal data and/or biosamples, and these activities are often associated with ethical issues. A systematic review was conducted to assess the proportion of publicly available online reports of the research activities undertaken on humans in Darfur between 2004 and 2012 that mention obtaining ethical approval and/or informed consent.

**Methods:**

This systematic review is based on a systematic literature search of Complex Emergency Database, ReliefWeb, PubMed), followed by a hand search for the hardcopies of the eligible reports archived in the Centre for Research on the Epidemiology of Disasters (CRED) in Brussels.

**Results:**

The online search showed that out of the 68 eligible studies, 13.2% (9) reported gaining ethical approval and 42.6% (29) that an informed consent was obtained from the participants. The CRED search included 138 eligible reports. None of these reports mentioned gaining ethical approval and 17 (12.3%) mentioned obtaining informed consent from their participants.

**Conclusions:**

The proportion of studies reporting ethical review and informed consent was smaller than might be expected, so we suggest five possible explanations for these findings. This review provides empirical evidence that can help in planning ethical conduct of research in humanitarian settings.

## Background

Armed conflicts are known to impact upon the physical and social structures of affected communities, mainly by forcing people to migrate to areas either within their country or outside it. These groups are referred to as internally-displaced persons (IDPs), and refugees, respectively [1]. A third category of those affected by armed conflict is known as the ‘host community’, which refers to the population that has received the refugees and/or the IDPs although they are not directly involved in the conflict.

The region of Darfur, west Sudan has a surface area of 510,888 km^2^, an area equal to that of France, and its pre-conflict population was estimated to be 6.7 million. In 1994, the region was divided into three states: North, South, and West Darfur as a part of the implementation of the federal system in Sudan.

Since 2003, armed conflict has been taking place in Darfur, mainly between the rebel groups (mostly non-Arabic speaking tribes) and the government of Sudan (GoS) or their allegedly aliened militias, known as Janjaweed. Other conflicted has been taking place between and among the different rebel groups, and between nomads and sedentary tribes [2]. As a result, the UN estimated that 2.3 million people were internally displaced within Sudan [3], and over 300,000 Darfuri became refugees in Chad, and around 24,000 in Egypt [4]. Such refugees may try to seek asylum or go on to other countries like Israel, where there is an estimated 1200 Darfuri refugees [5]. The IDPs are gathered in camps, which have their own local administration and receive humanitarian aid from the INGOs in their respective sectors. The main sectors include health, led by the World Health Organization (WHO), food and nutrition led by World Food Program (WFP), and education led by United Nations Children’s Fund (UNICEF). Other UN agencies distribute non-food items, like plastic sheets that the IDPs use to cover their mud-made ‘houses’ and tents.

The conflict in Darfur has attracted the attention of the international community since 2004, and has led to an influx of about 100 INGOs working in the region [1]. The number of humanitarian workers grew from more than 10,000 (of which more than 900 were international) in 2005 [6] to 17,100 in 2008 [7]. As part of their work, the humanitarian aid agencies conducted several activities that involved the collection of personal data and/or biological samples from those affected by the conflict, mostly in the form of household surveys and assessments. These surveys aim to assess the humanitarian impact of the conflict by looking for a set of epidemiological indicators, like morbidity, mortality, and malnutrition. For example, the Complex Emergency Database (CEDAT) records more than 800 mortality, nutrition and vaccination surveys that were undertaken in Darfur between 2004 and 2012 [8]. Moreover, Degomme suggests that the surveys that were undertaken in Darfur between 2003 and 2008 included more than 56,000 households, more than 100,000 children and more than 130,000 adults [9].

Some of the ethical issues related to research in war-torn areas have been discussed in the literature, such as the over-researching of particular conflict-affected populations [10], the vulnerability of refugees as research subjects [11], the need for an ethical code of conduct for research in humanitarian emergencies [1] and the refugees’ capacity to take an active role in research [12]. To address similar ethical issues, Ford and colleagues have emphasized the importance of considering the vulnerability of the researched communities, the need for the research to be conducted and the feasibility of doing such research in assessing the ethical aspects or research in conflicts [13]. However, there remains a need for greater in-depth systematic study of the ethical issues that were encountered and how they were managed by the researchers and the researching institutions.

### Rationale for this systematic review

To establish (or reform) the ethical oversight of research conducted during armed conflicts, the on-going related practices that can be described as research have to be described and discussed. These practices include the planning, the ethical review and the actual conduct of the humanitarian activities that can be considered as research. However, there is a lack of knowledge about the research management system in Darfur where ethical issues should have been anticipated and dealt with. Moreover, it is unclear whether such a system exists. The systematic review was thus needed to give an initial indication of how the ethical issues related to the studies conducted in Darfur were presented in the resulting reports.

There are many ethical issues related to the conduct of research that the mainstream research ethics guidelines have considered and set standards for [2]. These guidelines and standards were set to be always applicable, including the public health emergencies and humanitarian situations. Among them, two pillars are the need for ethics review and informed consent, although they may not be sufficient to ensure the ethical conduct and reporting of the research. To avoid publication of unethical research in a peer-reviewed journal, the ICMJE explicitly require clear statements from the authors that ethical approval for the execution of the reported study was obtained and consent was given by the participants, among other requirements [3]. Whether the reports of the studies conducted in Darfur would follow the same standards was not clear. Thus, we focused on only two ethical issues as an easily checkable proxy for a minimal consideration of ethical issues. The first was whether the published reports of the studies that were undertaken in Darfur mentioned that they had obtained ethical approval, and the second was if they mentioned obtaining informed consent from their participants.

These two issues were chosen only as examples of relevant ethical issues. Accordingly, the data extraction form was left open to the possibility of other ethical issues being recorded; for example, if the authors of the study disclosed any conflicts of interest or described how privacy or confidentiality was maintained, this could be mentioned in the results. The two examples were chosen because they were expected to be mentioned in the eligible reports. This expectation was based on two main assumptions. First, the main internationally-acknowledged research ethics guidelines, as well as those of Sudan [4], unanimously hold that any research that involves humans ought to obtain ethical approval [5] (for example, Guidelines 2 and 20 in the Declaration of Helsinki [6]; Common Rule, subpart A, especially articles 46.107, 46.108 and 46.109 ([7]), and that research participants should give voluntary informed consent (for example CIOMS General principles, guidelines 4–6 [8]. These requirements are meant to be followed regardless of whether the researcher intends to publish the research in a scientific journal. Second, these two issues are among the main requirements for publication of research that involves humans in medical journals [3]. Therefore, it is reasonable to expect them to be mentioned more often than other issues.

## Methods

### Selection criteria and literature search

This systematic review (SR) sought to include all studies published between 2004 and 2012 that involved the collection of human personal data and/or biosamples from the people of Darfur within or outside Darfur. These two criteria were not meant to provide an alternative definition of ‘research’, rather an attempt to avoid the complexity of finding an agreed-on definition of health-research, especially in the public health arena [9–11]. The criterion of data collection (with or without the collection of additional biological sample) is a necessary (though not sufficient) condition for any activity to be described as research. We acknowledge that the humanitarian activities include a systemic collection of data that are later analysed and utilized. Nevertheless, unlike pure (clinical) research that seeks generalizability, the humanitarian workers can use the collected data for other short-term, mission-specific, and organisation-oriented purposes.

Human personal data refers to any kind of information that could be used to identify a person or information pertaining to a person’s health-related conditions. These data include but are not limited to name, age, sex, address, and contact information. Biosamples refer to any human biological sample taken from Darfuri persons for purposes not solely related to their care, including but not limited to samples of tissue, blood, urine, and stool.

The SR included two main sources. The first was the reports that were publicly available online of the eligible studies published within the study period in English and/or Arabic with no limit to the participants’ group or the study methodology, hereafter referred to as the ‘online reports.’ The second were the results of a hand search of the hardcopies of the reports of the health-related studies conducted in Darfur during the study period archived in the CRED. The CRED’s archive contains a compilation of reports of surveys containing core data on the occurrence and effects of over 18,000 mass disasters all over the world, running from 1900 to the present [14]. These reports are received from the INGOs responding to the disasters [12] and include studies conducted in Darfur during the relevant time period, hereafter referred to as ‘CRED’s reports’ or the ‘CRED’s search’.

The purpose of the hand search of the CRED’s archive was to complement and validate the findings of the online systematic review. It involved a hand search of the reports of the studies that met the inclusion criteria for the online systematic review but were only available offline. The hand search was meant to complement the online search in case some reports were only available in hardcopy. It is also reasonable to believe that the hardcopies of the full reports may have included details about ethical issues not mentioned in the published reports and manuscripts available online, which are usually limited by word counts.

The study period was chosen with the aim of capturing all studies undertaken from the beginning of the influx of international aid agencies to Darfur in 2004, with the end of 2012 being the point at which it is reasonable to assume that any studies that had been completed would have been published. The eligibility criteria can be found in Table 1.

**Table 1:** Eligibility criteria used for screening, inclusion and exclusion of studies

	Included	Excluded
Topic	Any study that addressed any topic related to the health of the people of Darfur and involved the collection of personal data and/or biosamples from its participants was included, provided its full report or manuscript was retrievable from the online search and/or the CRED archive.	News, updates, political documents and retrospective studies analysing secondary data only
Types of studies and data items	Surveys, assessments, evaluations, situation reports and any study type that included the collection of personal data and/or biosamples directly from the participants or through reviewing records that contained their identifiable personal data	Infographics, manuals and guidelines, maps, news and press releases, and UN documents (e.g. legal documents and UN Security Council resolutions)
Types of participants	Darfuri people who were affected by the armed conflict, whether living inside or outside Darfur at the time of the study, whether IDPs, refugees or affected host communities	Studies on NGOs’ or GoS’ staff, general non-Darfur community, and studies on non-human participants
Types of interventions	Any study that was carried out on Darfuri persons during the study period, whether aimed at assessing the humanitarian impact of the crisis or not and regardless of whether it had a section or a statement on ethical review, ethical guidelines or consent	Studies that aimed at environmental or animal-related interventions
Settings	Any setting in which those affected by the Darfur conflict could be found, including but not limited to IDPs, refugee camps, and host communities	Any armed conflict setting outside Darfur
Types of publications and publication status	Any full report or manuscript that was retrievable from the online search or the CRED archive and published between 2004 and 2012 about findings from research that involved the collection of personal data and/or biosamples regardless of the purpose, the methodology, or the place of publication	Abstracts only, summary only reports, incomplete or inaccessible articles or reports, conference proceedings, meta-analyses, and reports on other activities that do not include the collection of human data and/or biosamples
Language of publication	English and Arabic	Reports published in any language other than English and Arabic
Publication date	1-1-2004 until 31-12-2012	Reports published before 1-1-2004 or after 31-12-2012

The Search terms that were used included a combination of MeSH terms, free text, and synonyms in order to capture as many of the relevant publications as possible. The search terms that were used were: Humanitarian aid, Assessment, Surveys, Nutrition*, Darfur, Sudan, Refugees, Camps, Internally displaced persons (IDPs), Child, United Nations, Non-Governmental Organization, Ethics Committees, ethic*, and Informed Consent. We have used search strings that included OR between all the keywords except for Darfur, which was proceeded with AND to minimize the appearance of studies that were not conducted in Darfur. For example, to include the nutritional surveys that assessed malnutrition in children, the search string was (“*Nutrition*” OR “children” AND “survey” OR “assessment” AND “Darfur”).

**Fig. 1:**
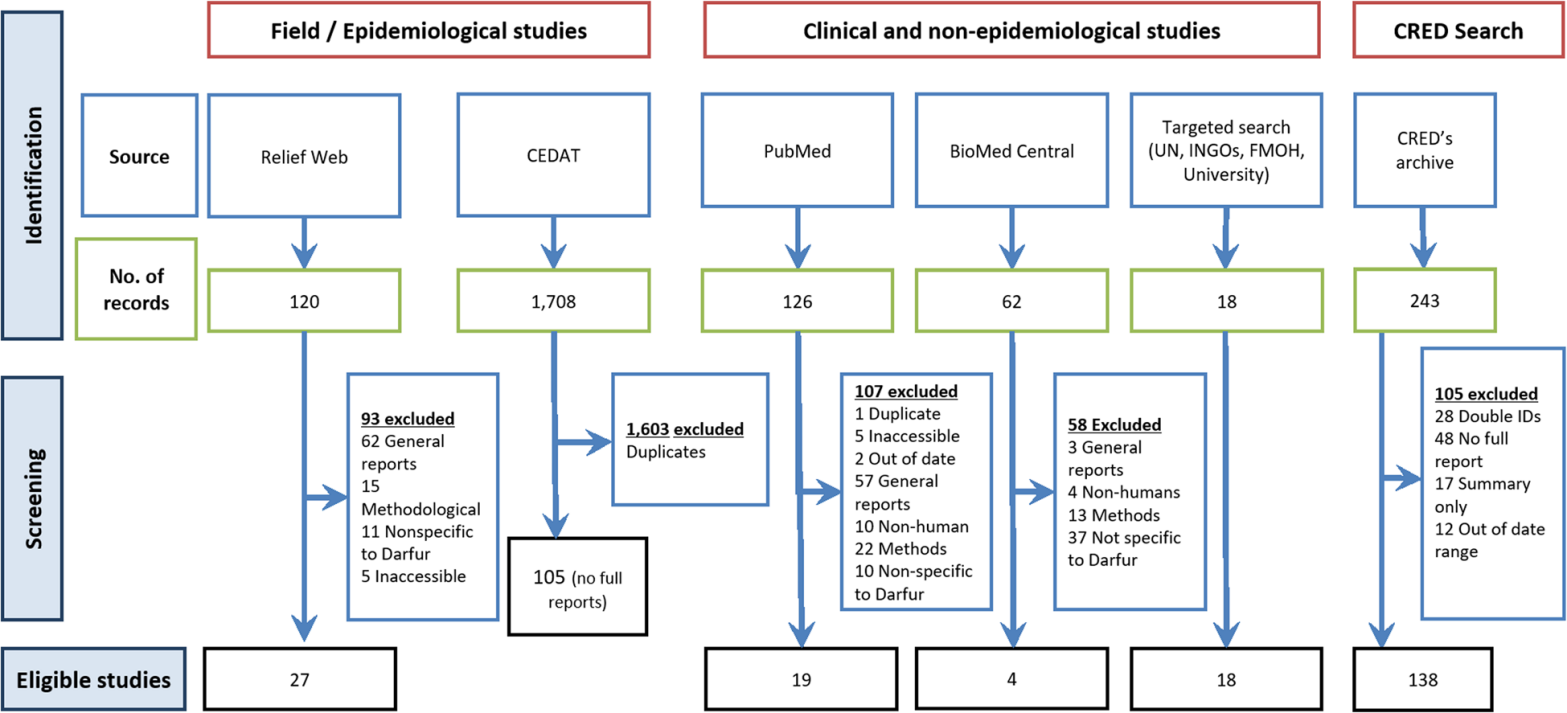
PRISMA 2009 Flowchart for online and CRED search. There were two main sources for search for studies. The full reports of the humanitarian-related studies were searched in ReliefWeb (http://reliefweb.int), which is a specialized digital service of the United Nations Office for the Coordination of Humanitarian Affairs (OCHA). 27 studies were eligible in ReliefWeb. Complex Emergency Database (CEDAT) was only used for secondary analysis, as it does not provide full text reports so none of its reports was eligible for inclusion. Second, the clinical and non-epidemiological studies were searched in PubMed, BioMed Central, where 19 and 4 studies, respectively were considered eligible, and targeted search in the websites of the main international humanitarian organizations and the Sudanese federal ministry of health. Targeted search resulted in 18 eligible studies

The search included two main streams of searching (Fig. 1). Firstly, the operational and humanitarian-related studies were retrieved from the Complex Emergency Database (CEDAT, http://cedat.be/), which is managed by the Centre for Research on the Epidemiology of Disasters [15]; Complex Emergency Database [16]. The eligible studies’ titles were then searched in ReliefWeb (http://reliefweb.int), which is a specialized digital service of the UN Office for the Coordination of Humanitarian Affairs [1]. Secondly, the clinical and non-epidemiological studies were searched in PubMed and Biomedcentral (Table 2).

**Table 2:** Sources of specialized search to retrieve the full-text reports or manuscripts when not found in ReliefWeb

a. United Nations Sudan Information Gateway (UNSIG): http://www.unsudanig.org has weekly ‘Humanitarian Action in Darfur’ reports since 2006,	
b. WHO’s IRIS (World Health Organization Institutional Repository for Information Sharing): https://apps.who.int/iris/	
c. Médecins Sans Frontières (MSF) field research database: https://fieldresearch.msf.org/msf/	
d. Google Scholar	

The main source of the full-text reports for the screened studies was ReliefWeb (https://fieldresearch.msf.org/msf/); where the full report was not available, the websites of the respective humanitarian organization was searched. Google scholar was the main source to search for grey literature

Each of the eligible studies was appraised based on two main indicators: whether there was mention of obtaining an ethical approval and whether there was mention of obtaining an informed consent from the participants.

Data extraction was conducted using a pre-designed data extraction form (http://www.tfaforms.com/271050) that was piloted in a sample of studies and then corrected and modified in order to improve its ability to capture as many relevant details about the included studies. The review depended solely on the information available in the published online sources of the eligible manuscript/report. The authors of the eligible reports were not contacted to request information not found in the published report.

### Assessment of risk of bias in included studies

Two potential reporting biases may have accompanied this systematic review: publication bias and access bias. In terms of publication bias, the included studies were likely to have been intended to meet humanitarian needs and were not usually prepared for academic purposes. Thus, these data/biosample collection activities are often not reported in the standard format for reporting research studies. Accordingly, the surveying agencies might not follow the standard requirements for publication in peer-reviewed journals, including the requirement to mention having obtained ethical approval or informed consent from participants. This bias was addressed by contacting the authors of the included studies where possible to cross-check whether the included study had received formal ethical approval and, if not, to explain the reasons for not having received approval.

Second, access bias could result from the availability of some of the eligible studies on subscription-based databases that required institutional affiliation that we did not have. This bias was addressed by searching in multiple sources, including hand searching. For example, only 68 studies were found in the online search, while the hand search in the CRED archive included 138 additional studies.

## Results

Out of the 2034 retrieved studies through the search, 68 studies were eligible for this review. Figure 1 summarizes numbers of studies screened, assessed for eligibility, and included in the review, with reasons for exclusions at each stage. Notably, all eligible reports were in either in Arabic or English, i.e. no report was excluded based on its language of publication. Table 3 reports the characteristics of the eligible studies included in the systematic review.

**Table 3:** Main characteristics of the studies included in the systematic review (*N* = 68)

	Characteristics of the included studies	CRED (*N* = 138) (%)	Online (*N* = 68) (%)
Study theme	Children’s illnesses (including diarrhoea/ Acute Respiratory Illness)	48 (34.8)	11 (16.2)
	Clinical conditions (including AIDS, malaria, genetic diseases)	4 (2.9)	16 (23.5)
	Immunisation	119 (86.2)	10 (14.7)
	Mental health issues	0 (0.0)	8 (11.8)
	Methodological, organisational issues	0 (0.0)	2 (2.9)
	Morbidity	91 (65.9)	12 (17.6)
	Mortality	124 (89.9)	18 (26.5)
	Nutrition and food security	130 (94.2)	28 (41.2)
	Other	34 (24.6)	24 (35.3)
	Violence and gender-based violence (GBV), including rape	0 (0.0)	14 (20.6)
	Water, Sanitation, Hygiene (WASH)	37 (26.8)	8 (11.8)
	Women/Maternal/Reproductive Health	2 (1.4)	46 (67.6)
Type of the main surveying agencies	UN agency	27 (19.6)	28 (41.2)
	Independent researchers	0 (0.0)	27 (39.7)
	INGO	119 (86.2)	23 (33.8)
	Governmental body	46 (33.3)	14 (20.6)
	International (bilateral) agency	9 (6.5)	3 (4.4)
	NNGO	2 (1.4)	2 (2.9)
Data collection methods and tools	Questionnaires - Interviews (including verbal autopsy)	135 (97.8)	50 (73.5)
	FGDs	10 (7.2)	23 (33.8)
	Anthropometric measures	128 (92.8)	11 (16.2)
	Blood/serum sample	2 (1.4)	11 (16.2)
	Review of medical records	11 (8.0)	10 (14.7)
	Review of non-medical reports	116 (84.1)	8 (11.8)
	Direct observations (including observing oedema)	106 (76.8)	7 (10.3)
	Others	7 (5.1)	4 (5.9)
	Urine/stool sample	0 (0.0)	3 (4.4)
	Other body sample	0 (0.0)	1 (1.5)
Sampling techniques	(Multi-stage) cluster sampling	137 (99.3)	36 (52.9)
	Convenience/targeted (Non-random		27 (39.7)
	Not applicable		2 (2.9)
	Not mentioned		2 (2.9)
	Other	1 (0.7%)	1 (1.5)
	Semi-random sampling		1 (1.5)
	Systematic/random sampling		6 (8.8)

### Overview on the studies eligible for inclusion

The proportion of eligible studies is considerably low compared to the actual research activity over the study period, at least compared to CEDAT. There are 803 mortality and nutritional surveys undertaken in Darfur (2003–2012) whose results are reported in CEDAT (http://cedat.be/). The peak for the conduct of both mortality and nutritional surveys was between 2005 and 2007, concomitant to the influx of international humanitarian aid agencies. In 2005 alone, more than 150 nutritional surveys and 50 mortality surveys were undertaken in Darfur [13].

The monthly distribution across the year shows that there was on average 2.8 studies undertaken per month (range 1–5). More than half (39; 57.4%) of the studies included in this review were studies aimed at assessing the humanitarian impact of the conflict on the people of Darfur through a set of epidemiological indicators. Twenty-eight (41.1%) articles, mostly published in peer-reviewed journals (26, 34%), were not only concerned with the humanitarian impact, but also specific conditions like hepatitis, or genetics. There was only one registered randomized controlled trial (RCT) on Darfuri refugees living in Cairo.

The main themes and indicators of the included studies are summarized in Table 3.

All of the 18 retrieved household surveys studied more than one epidemiological theme, for example mortality surveys would also study morbidity or food security. Therefore, the percentages of different themes sum up to more than 100%.

### Type of collected data and data collection methods in the included studies

In both the online and CRED searches, the most commonly used data collection tools were questionnaires (135; 97.8% and 50; 73.5% respectively). However, in CRED studies, the use of anthropometric measures (128; 92.8%) such as height and weight and the review of non-medical reports such as vaccination cards (116; 84.1%) were mentioned more frequently than in the online studies (23; 33.8% and 11; 16.2%, respectively). The use of anthropometric measures is common in nutritional surveys, and review of vaccination cards helps in minimising recall bias and in validating the answers of the carer in surveys involving children.

The mention of the use of FGDs was higher in the online studies (23, 33.8%) than in CRED studies (10; 7.2%). Similarly, taking biosamples (mainly blood (whole or serum) (11; 16.2%) and urine/stool (3; 4.4%)) was mentioned more in the online studies (15; 22.1%) than in the CRED studies (2; 1.4%) (Table 3).

The most commonly used population sampling technique in both the online and the CRED studies was two-stage cluster population-proportional sampling (36; 52.9%, and 137; 99.3%, respectively).

### Target populations and locations of the included studies

The most commonly used sampling techniques were two-stage cluster population-proportional sampling and convenience or targeted sampling (36; 52.9% and 27; 39.7%) respectively.

The main target populations for the included studies were IDPs (49; 72.1%), host communities (28; 41.2%) and refugees (13; 19.1%). Most of the included studies were conducted in IDP camps (39; 57%), affected community areas (31; 46%) and refugees’ locations (usually camps) (8; 12%).

The three Darfur states of West, North, and South Darfur were targeted almost equally by the included studies, 38 (55.9%), 34 (50%), and 34 (50%), respectively. Six studies (8.8%) were undertaken in neighbouring Chad on Darfuri refugees.

### Mention of ethical review

**Fig. 2:**
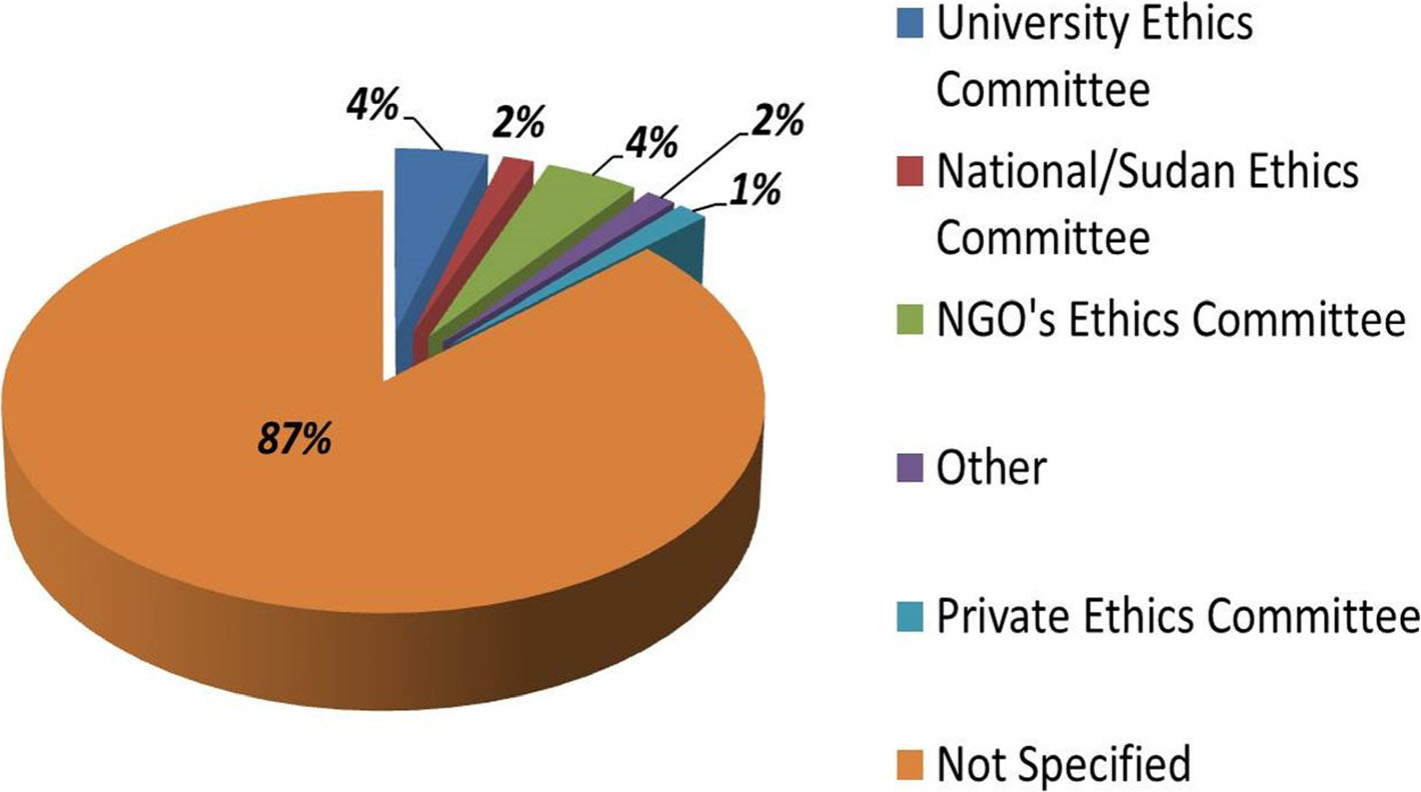
Sources of ethical approval as mentioned in the studies included in the systematic review (*N* = 68). Out of 68 eligible studies included in this review, only 9 (13%) mentioned that they were ethically approved. Three studies (4%) were approved by a university ethics committee, three studies (4%) were approved an NGO’s ethics committee, while only one study mentioned to be approved by the national research ethics committee (NREC) of Sudan

None of the reviewed CRED studies mention seeking or obtaining ethical review or approval. The online search revealed that nine studies (13.2%) mentioned that they had obtained ethical approval. Of these, three studies were approved by a university ethics committee, three were approved by the surveying INGO’s ethics committee, and only one study was reviewed by the Sudanese NREC. Eight of these nine studies (89%) were retrieved from peer-reviewed journals, while one study was retrieved from the website of the federal ministry of health in Sudan (Table 4 and Fig. 2).

**Table 4:** Characteristics of the studies mentioned obtaining ethical approval

	Study A	Study B	Study C	Study D	Study E	Study F	Study G	Study H	Study I	Study F
Type	Article	Article	Report	Article	Article	Article	Article	Article	Article	Article
Who conducted it?	- Independent authors	- INGO - UN Agency - Government	- Governmental	- Independent authors	- Independent authors	- INGO - Independent authors	- INGO - Independent authors	- Independent authors	- Independent authors	- INGO - Independent authors
Type of study/ article	Non-randomized Non-epidemiological research	Non-randomized Non-epidemiological research	Household (Multi-Indicator) Survey Other	Non-randomized Non-epidemiological research	Household (Multi-Indicator) Survey	- Non-randomized Non-epidemiological research	- Mortality survey - Nutrition/food assessment	- Household (multi-indicator) survey	- Household (multi-indicator) survey	- Non-randomized Non-epidemiological research
Retrieved from	Peer-reviewed journal	Peer-reviewed journal	FMOH website	Peer- reviewed journal	Peer-reviewed journal	- Peer-reviewed journal - INGO website	- Peer-reviewed journal	- Peer-reviewed journal	- Peer-reviewed journal	- Peer-reviewed journal - INGO website
Themes and indicators	- Women/ maternal health - Mental health issues - Violence (GBV/rape)	- Clinical/ medical conditions	- Child health/rights - Malaria - Women/ maternal health	- Human rights - Violence (GBV/rape)	- Violence (GBV/rape)	- Mental health issues	- Mortality - Nutrition and food security - Violence (GBV/rape)	- Other (poverty)	- Human rights - Women/ maternal health - Mental health issues - Reproductive health	- Mental health issues
Type of data collected	- Primary identifiable data - Primary non-identifiable data	- Primary identifiable data - Primary non-identifiable data	- Primary non-identifiable data	- Secondary identifiable data - Secondary non-identifiable data	- Primary identifiable data - Primary and secondary non-identifiable data	- Primary identifiable data - Primary non-identifiable data	- Primary identifiable data - Primary non-identifiable data	- Primary identifiable data - Primary and secondary non-identifiable data	- Primary identifiable data - Primary non-identifiable data	- Primary identifiable data - Primary non-identifiable data
Were human specimens collected?	No	Yes	Yes	No	No	No	No	No	No	No
Data/ biosamples collected	- Questionnaires - Interviews	- Questionnaires - Urine/stool samples	- Questionnaire - Interviews - Blood/serum samples	- Review of medical records	- Review of nonmedical reports - Questionnaires - Interviews	- Review of medical records - Questionnaires	- Questionnaires - Interviews - Anthropometric measures	- Questionnaires - Interviews - FGDs	- Questionnaire - Interviews	- Review of medical records - Questionnaires
Study area and population	- Affected community area	- General community	- General community	- NGO/desk review	- Refugee camp	- Health facility	- IDP camps - Affected community area	- IDP camp - Affected community area - NGO/desk review - General community	- IDP camp	- Health facility
Darfur Region(s) included in the study	- West Darfur - North Darfur - South Darfur	- South Darfur	- West Darfur - North Darfur - South Darfur - Other Sudanese States	- South Darfur	- Chad	- West Darfur	- South Darfur	- West Darfur	- South Darfur	- West Darfur
Sampling technique	- Convenience/ targeted (non-random)	- Semi-random sampling	- (Two-stage) cluster sampling	- Other: retrospective review and analysis of medical records of victims of torture	- (Two-stage) cluster sampling	- Convenience/ targeted (non-random)	- (Two-stage) cluster sampling	- (Two-stage) cluster sampling	- Systematic/ random sampling	- Convenience/ targeted (non-random)
Source of ethical approval	- Academic ethics committee	- Other	- National Research Ethics Committee	- NGO’s ethics committee - Academic ethics committee	- Private ethics committee	- NGO’s ethics committee	- NGO’s ethics committee	- Academic ethics committee	- Private ethics committee	- NGO’s ethics committee
Name of the ethics committee	- AUW ethics committee	- The study was approved by SMoH, followed by the local health authority.	- National Research Ethics Committee	- The MSF Ethical Review Board	- American Bar Foundation’s institutional review board	- The MSF Ethical Review Board	- MSF and Epicentre	- University College London	- The Western Institutional Review Board	- The MSF Ethical Review Board
Participant consent obtained?	Yes	Yes	Yes	Not stated	Yes	Yes	Yes	Not Stated	Yes	Yes
How was it obtained?	Written	Verbal	Written	Not stated	Verbal	Verbal	Verbal	Verbal	Verbal	Verbal

Study A: Exposures to war-related traumatic events and post-traumatic stress disorder symptoms among displaced Darfuri female university students [18]

Study B: High prevalence of urinary schistosomiasis in two communities in South Darfur: implication for interventions [19]

Study C: Malaria Indicator Survey Northern States of Sudan – October-November 2009 [20]

Study D: Medical evidence of human rights violations against non-Arabic-speaking civilians in Darfur: a cross-sectional study [21]

Study E: Racial Targeting of Sexual Violence in Darfur [22]

Study F: Mental health treatment outcomes in a humanitarian emergency: a pilot model for the integration of mental health into primary care in Habila, Darfur [23]

Study G: Mortality and Malnutrition Among Populations Living in South Darfur, Sudan Results of 3 Surveys, September 2004 [24]

Study H: Child Poverty in an Emergency and Conflict Context: A Multidimensional Profile and an Identification of the Poorest Children in Western Darfur [25]

Study I: Basic Health, Women’s Health, and Mental Health Among Internally Displaced Persons in Nyala Province, South Darfur, Sudan [26]

### Mention of informed consent

The studies that did not mention obtaining consent were 121 (58.7%) and 39 (18.9%) in number in the CRED and online searches, respectively; while the studies that mentioned obtaining informed consent from their participants in the online search numbered 29 (42.6%) and in the CRED search 17 (12.3%).

Approximately one-third of the studies that mentioned obtaining informed consent (*N* = 29) were found by the online search (9; 31%). Informed consent was mostly obtained verbally in the results of both the online and CRED searches (18; 26.5%, and 13; 9.4%, respectively).

### Communication with authors

Following the online search, 60 authors’ emails were retrievable from either the published reports or the internet. An email was sent to each author to introduce this review and to invite them to fill in an online form (Appendix 11). Only eight authors filled in the form. None of the answers provided led to any modifications or corrections in the results of the online review. The CRED search did not result in any contact details additional to those found online.

## Discussion

Overall, the results presented an answer to the main search question, which addressed two variables regarding the publicly available online reports of the research activities undertaken on humans in Darfur between 2004 and 2012, which are the proportion of mentioning obtaining ethical approval and/or the mention of informed consent. In this section, we are providing a general overview of these results followed by a number of possible explanations to help in understanding them. These possibilities were discussed using the relevant literature, and when possible, by quotes from the studies themselves.

### General overview on the findings

In the 68 included studies, the most common methodology was that of household multi-indicator surveys, which were mostly done by the UN agencies and/or other INGOs (51; 75%) and focused on mortality, morbidity and nutrition. They are key elements to planning, implementing and assessing humanitarian interventions in Darfur, although other conditions were also studied like hepatitis, malaria, and HIV/AIDS, and genetics. There was only one retrievable RCT on Darfur refugees in Cairo. This is unsurprising as RCTs generally need more stable settings, as well as technical and logistic requirements that are hard to provide in a conflict setting. Moreover, RCTs are unlikely to be methodologically useful to answer the kinds of research questions raised in conflict settings.

The degree to which household questionnaire-based surveys were so dominant amongst the studies in this review can be used to understand the overall findings. For example, the INGOs may have considered such research activities as ‘low risk’ studies that did not include invasive measures, and therefore ought to be exempted from ethical review. In addition, different agencies may have different policies related to their research practices, including different levels of ethical review depending upon the method and/or risks involved. These hypotheses and possibly others need to be tested in future work.

### Mention of ethical review

As none of the CRED studies mentioned their ethical approval status, the following focuses only on the studies retrieved from the online search. The majority of the studies (59; 86.6%) did not report whether they had gained a favourable ethical opinion. This does not necessarily mean that ethical review was not sought or that they did not receive a favourable opinion where one was sought. However, if we assume that the findings from the studies available for review reflect the general picture of all studies conducted in Darfur during the relevant period, we can hypothesise about why the two ethical features (ethical approval and informed consent) were only mentioned so rarely.

These possibilities include the following:

- *Possibility one: These studies were exempted from ethical review.*

The plausibility of this option is supported by a statement that was found in one of the studies included in this review. The Crude Mortality Survey, led by the WHO and jointly conducted by other UN agencies and the GoS, stated that “WHO guidelines do not require ethical review for retrospective surveys during humanitarian emergencies …” [17].

This statement reflects that at least some of the WHO surveys during emergencies are exempted from ethical review, and this is likely to include the surveys included in this review which were led or actually undertaken by the WHO (7; 10.3%). However, it is not possible to derive a general conclusion from a statement mentioned in one study.

Other possible reasons for exemption from ethical review are provided by MSF’s ethics committee including where the research involves “routine programme implementation and assessment related work” [27]. It is not clear in the guidelines, whether these ‘assessment-related work’ included the collection of personal data and/or biosamples or not. This exemption is unlikely to have been applied in the studies included in this systematic review, whose main inclusion criterion was the prospective collection of personal data and/or biosamples. Moreover, it would be expected than even when these surveys are not done primarily for research purposes may not need ethical approval; yet they would have complied with an essential ethical requirement such as consent.

- *Possibility two: Mentioning ethical review was not required*

The majority of these studies (39; 57.4%) were conducted by humanitarian aid agencies. As a part of sharing experiences and lessons learned among these agencies, they could be more interested in sharing their methodologies and findings than sharing other details. Therefore, the published reports would be expected to focus more on the methodological details and results than items related to the ethical issues. However, even if this is true of the studies published in disaster-specific databases where mentioning ethical review or consent is not a requirement for publishing a study report, it should not apply to the one third of studies (26, 34%) that were published in peer-reviewed journals. In the latter, “authors should indicate whether the procedures followed were in accordance with the ethical standards of the responsible committee” [28]. The discrepancy between the 34% of studies that were published in peer-reviewed journals and the 13.2% that mentioned being ethically reviewed suggests that studies were published in peer-reviewed journals without stating their status of ethical approval in the published papers. Further contact with the authors and the journal editors is needed to exclude the possibility that the status of ethical approval was made available to the editors (e.g. in covering letters accompanying a manuscript) but not included in the published articles.

- *Possibility three: Ethical review was considered by the researchers as if granted*

This possibility assumes that the studies done by the organisation were assumed to have been ethically approved, and so the researchers did not have to mention this approval in their published reports. This possibility could be supported by other findings in the study, as one third of the ethically approved studies were either reviewed by the MSF ethics committee or exempted by them as they had met prior established criteria set out in its ethical guidelines. It could be assumed that MSF applies its ethical standards to all its surveys [29]. Moreover, MSF has its own criteria for exempting some of the field research from ethical review [27], which could explain why some of its studies reported ethical review while others did not.

- *Possibility four: Pre-approved proposals*

This is an alternative approach to ethical review that is based on ethically reviewing and approving ‘ready-made’ generic study protocols of ‘emergency research’ when the research needed to be conducted in an urgent and timely manner, i.e. it cannot wait for full ethical review. This approach has been suggested for research to be conducted during pandemics [18] and is adopted by the MSF ethics committee in very special circumstances [27]. In the context of this systematic review, this could mean that one or more of the included studies may have been held to be ‘emergency research’ that was a part of wider research whose protocol was previously ethically approved. However, the findings of this systematic review do not provide any evidence of that, as the reviewers did not assess whether these studies met the criteria for exemption stated in the MSF guidelines. We could not find any finding in this review to support this possibility. Additionally, the MSF guidelines clearly states that exemption by MSF Ethics Board “does not exempt MSF to comply with regulatory requirements in the country from where the data originate …” and “local ethical review may still be required.” [28] Moreover, the MSF ERB still requires the ethical approval of the final protocols that used pre-reviewed generic protocols. Thus, the studies under this category should have mention of ethical approval.

- *Possibility five: The ethical review was not part of the template used*

At least for the studies retrieved from the CRED search, the patterns and formatting used for reporting were very similar, as though they used a common template. These similarities applied to the methodologies and the reporting of the results. For example, multi-stage cluster sampling was used by almost all of the CRED studies reviewed (137; 99.3%) and more than half of those found online (36; 52.9%). Moreover, many of the reports used exactly the same wording to describe the sampling procedure.

This possibility can also be supported by the finding that the studies conducted by one INGO mentioned ‘Ethical Considerations’ using exactly the same words and structured under exactly the same bullet numbering [8, 9]. This is particularly significant if other INGOs also use a template. Theoretically, changing the template that such organisations use to report their studies may change the extent of inclusion of ethical considerations in future studies. For example, if a template included a section on ‘informed consent’ or ‘ethical approval’, then those using it would be likely to include more details about these aspects.

### Discussion of the studies that mention being ethically approved

Given that almost all of the studies included in this review were conducted in the Sudan (apart from the trial that was conducted in Cairo); it would be expected to have them reviewed and approved by a Sudanese ethics committee. The main body responsible for such review is the National Research Ethics Committee, as stated in the Public Health Act [16] and the Sudanese national guidelines [4]. Given the complexity of the setting in which they were conducted, it would be expected that other alternatives would be considered. However, the studies that mention being ethically approved do not have much in common, but they do appear to reflect the general trend of the other studies. One significant exception is that almost all these articles were published in peer-reviewed journals [19–21, 29–34] (Table 4). Additionally, there are two points worth noting. First, MSF’s procedures on the ethical review of its field surveys [17] were the only INGO ethics-related oversight mechanism mentioned in the studies included in this review. Other NGOs might have their ethics committees and procedures, but they were not mentioned.

Second, there were only two studies ethically approved in Sudan. One was reviewed by a Sudanese university’s ethics committee [19], while the other was the only study that was reviewed and approved by the NREC [21]. Both committees are in Khartoum, not Darfur. The Sudanese research ethics guidelines defines research as “any social science, biomedical, behavioural or epidemiological act that entails systematic collection or analysis of data with the intent to generate new knowledge, in which human being are involved” [4]. With such a broad definition of research, it is reasonable to assume that the studies included in this review would count as research that needs to be ethically reviewed. In addition, all research studies that have non-Sudanese researchers should have been reviewed by the NREC if the Sudanese research ethics guidelines were followed [4, 16]. However, this finding could be for other reasons, such as a lack of ethics review capacity in Darfur, and perhaps Sudan as a whole, as other studies have already concluded [27, 29].

### Mention of consent

In the online search, more studies mentioned that they obtained consent (29; 43%) than mentioned that they had been ethically approved (19; 13.2%). More studies in the online search than in the CRED search (17; 12.3%) mentioned obtaining consent. The former finding could be partially explained by the fact that most of the included studies were household-based studies which used similar methodologies that were described in detail and made available to humanitarian aid workers to use [17, 27]. These methodologies are described in common guides used by the researchers in these agencies. These guides usually mention a section on ‘informed consent’ under the ‘methodology’ section, so those who use these templates consider obtaining consent a part of the methodology. This assumption could be supported by the finding that some commonly used template guides mention obtaining consent from participants without mentioning other issues related to ethical review [17, 29]. Therefore, those who follow these guides would only mention what these guides contain, which is consent and not ethical approval.

As might be expected, most participants’ consent was obtained verbally (18; 27%), which is more feasible than obtaining written consent, given the culture of Darfur where people do not like to or cannot sign papers.

The finding that consent was mentioned in more of the online studies than the CRED studies has two possible explanations. First, there is more variation in the studies found in the online search, which included publications in peer-reviewed journals in addition to epidemiological field reports. It is more likely to find consent mentioned in an article published in a peer-reviewed journal than in household surveys that are mainly shared for their epidemiological findings. Second, most of the CRED studies were produced by a relatively limited number of organisations whose main interest is the field-related details, namely the results and survey methods. In contrast, the online studies included studies done for non-humanitarian purposes by non-humanitarian researchers who may follow different reporting formats. Also, the NGOs might have used template guides of survey methodologies that did not include or did not emphasise the mention of consent. For example, consent is built into the first part of the standard survey template and is considered a routine that it is not considered worth mentioning on its own.

The reports that mentioned that informed consent was obtained from participants did not describe how this was achieved. However, there is a point worth noting in relation to the content and structure of the informed consent sheets that were found in a few cases attached to the studies that mentioned obtaining consent from the participants. Despite the variability in the requirements for obtaining consent to be considered ethically valid, there is arguably a common criterion of appropriate disclosure of information to the participant so that she or he is considered ‘informed’. The following examples suggest that this criterion was at least sometimes not met; assuming actual practice was guided by these statements.

#### Example 1: Consent from a food security and nutritional assessment survey [21]

“Consent: We are conducting a survey on the nutrition and food security of your family. I would like to ask you some questions about your family and we will also weigh and measure your children who are younger than 5 years of age. The survey usually takes about one hour to complete. Any information that you provide will be kept strictly confidential and will not be shown to other people. This is voluntary and you can choose not to answer any or all of the questions if you want; however, we hope that you will participate since your views are important. Do you have any questions? May I begin now? YES______ NO______”

#### Example 2: Consent from a household health survey [30]

“We are a team from the Sudan Household Health Survey that is concerned with family health and education. We would like to talk to interview you for about 45 minutes. All the information we obtain will remain strictly confidential and your answers will never be identified. During this time, I would like to speak with the household head and all mothers or others who take care of children in the household. MAY I START NOW? If permission is given, begin the interview.”

Finally, most of the CRED studies were produced by a relatively limited number of organisations whose main interest is the field-related details, namely the results and survey methods. In contrast, the online studies included studies done for non-humanitarian purposes by non-humanitarian researchers who may follow different reporting formats. Also, the NGOs might have used template guides of survey methodologies that did not include or did not emphasise the mention of consent. For example, consent is built into the first part of the standard survey template and is considered a routine that it is not considered worth mentioning.

## Conclusions

This review has presented the findings of one of the first empirical published works to explore the area of ethical considerations in the conduct of research during armed conflicts.

We have looked for only two examples of these ethical considerations: the mention of obtaining ethical approval and the mention of obtaining informed consent. We have found that the proportion of studies reporting ethical review and informed consent was smaller than might be expected. The reasons for this result could not be concluded on the basis of this review. However, we think that these findings can present a baseline empirical indicator to a potential gap in either obtaining these two ethical requirements or in reporting them. More work is needed to determine whether reported practice mirrors actual practice and, if so, why and whether this apparent deviation from research ethics norms is justified.

## Limitations

The findings of this systematic review are subject to three limitations. Firstly, the conclusions of this review are based on the data reported in the reports/manuscripts of the included studies, which may not be accurate. A satisfactory level of precision could not be confidently attained, even after searching offline resources and communicating with the authors and the surveying institutions, given the limited feedback received from the authors. Secondly, the status of ethical approval and informed consent were not always required to be included in the published versions of the study reports. The publication requirements vary depending on the policy of the surveying agency or the publisher, so comparing various types of reports may not be consistent. Lastly, an important limitation lies in the fact that the reports included in this study were significantly lower in number compared to what is known about the amount of research undertaken in Darfur during the relevant period. As mentioned earlier, CEDAT estimates that more than 800 surveys were undertaken in Darfur between 2004 and 2012 (CEDAT, 2013).

## Data Availability

All the data collected during the doctoral project, from which related to this systematic review is a part are stored in the servers of the University of Birmingham, as per the University’s regulations. Data are however available from the authors upon reasonable request after gaining the University’s permission.

## Abbreviations

CEDAT: Complex Emergency Database
CRED: Centre for Research on the Epidemiology of Disasters
ERB: Ethical Review Board (of MSF)
FMOH: Federal Ministry of Health, aka National Ministry of Health
GoS: Government of Sudan
IDPs: Internally Displaced Persons
INGO: International Non-Governmental Organisation
MSF: Médecins Sans Frontières
NGOs: Non-governmental organisations
NREC: National Research Ethics Committee
SMOH: State Ministry of Health
UNICEF: United Nations Children’s Fund
WFP: World Food Programme
WHO: World Health Organisation
